# Switching to alternative tobacco products during unsuccessful smoking quit attempts is not linked to improved quit intention or interest

**DOI:** 10.1186/s13722-026-00674-2

**Published:** 2026-05-25

**Authors:** Julia N. Soulakova, Lisa J. Crockett

**Affiliations:** 1 Department of Population Health Sciences, College of Medicine, University of Central Florida, 6900 Lake Nona Blvd., Orlando, FL 32827 USA; 2 Department of Psychology, University of Nebraska-Lincoln, 315 Burnett Hall, Lincoln, NE 68588-0308 USA

**Keywords:** E-cigarettes, Evidence-based cessation aids, Nicotine pouches, Smokeless tobacco, Smoking and cessation behaviors

## Abstract

**Background:**

Among adults who smoke cigarettes daily, attempts to quit smoking may involve using alternative tobacco products (ATPs) or evidence-based cessation aids, such as Nicotine Replacement Therapy (NRT). This study examined use of ATPs and aids during the last serious smoking quit attempt as well as quit intentions and interest in quitting among adults who smoke daily.

**Methods:**

This cross-sectional study utilized the 2022-23 Tobacco Use Supplement to the Current Population Survey data collected for U.S. adults (18+; *n* = 3,038 for using an ATP or aid, *n* = 2,962 for quit intention, and *n* = 3,000 for interest in quitting). Statistical methods accounted for the complex survey sampling design, e.g., multiple logistic regression models with balanced repeated replication for variance estimation.

**Results:**

Among the ATPs considered, switching to e-cigarettes (20.93%) or nicotine pouches (5.46%) was most common. Switching to cigars/pipes (adjusted Odds Ratio (aOR) = 2.38, 95% CI = 1.26–4.47), smokeless tobacco (aOR = 2.92, 95% CI = 1.61–5.30), or using NRT (aOR = 1.50, 95% CI = 1.13–1.99) was associated with switching to e-cigarettes. Switching to cigars/pipes (aOR = 3.01, 95% CI = 1.37–6.62), e-cigarettes (aOR = 1.74, 95% CI = 1.03–2.93), smokeless tobacco (aOR = 7.33, 95% CI = 3.42–15.72), or using NRT (aOR = 6.34, 95% CI = 3.79–10.61) was associated with switching to nicotine pouches. Among adults who used an ATP or aid, quit intention was lowest among those who switched to cigars/pipes (63.90%) and highest among those who used a digital tool or program (84.99%). Switching to any ATP was not associated with quit intention or interest in quitting. In contrast, use of any aid, except group counseling or support groups, was positively associated with quit intention, and use of any aid was associated with greater interest in quitting (all p’s < 0.05).

**Conclusions:**

The study highlights the emerging role of e-cigarettes and nicotine pouches as alternative quitting strategies, although no evidence was found that using these ATPs is associated with improved quit intentions or interest in quitting. In addition, the study demonstrates that evidence-based cessation aids continue to be underutilized. Further research is needed to elucidate the role of ATPs in smoking cessation alongside evidence-based cessation aids, as well as their potential for harm reduction among adults who smoke heavily.

**Supplementary Information:**

The online version contains supplementary material available at 10.1186/s13722-026-00674-2.

## Background

Adults who attempt to quit smoking use a variety of aids, including evidence-based methods such as Nicotine Replacement Therapy (NRT), which increases the likelihood of quitting [1–3], as well as alternative tobacco products (ATPs, defined as tobacco products other than regular cigarettes) that are not considered evidence-based cessation aids. Studies conducted in the U.S. show that e-cigarettes are most commonly used ATPs during quit attempts [4], while the NRT is the most commonly used evidence-based cessation aid [2, 5, 6]. Although fewer adults use e-cigarettes than NRT while attempting to quit smoking, switching to e-cigarettes (from regular cigarettes) is relatively common [5]. For example, in 2020, among U.S. adults who smoked daily or had quit within the past two years, 22.1% reported switching to e-cigarettes and 29.5% reported using NRT during their last quit attempt [5]. Likewise, analysis of longitudinal data collected in 2013-17 showed that 22.8% of adults who recently quit smoking switched to e-cigarettes during the quit attempt; however, this switching was not associated with preventing relapse [7]. A systematic review reached a similar conclusion, indicating that switching to e-cigarettes to quit does not facilitate smoking cessation [8]. However, two recent large-scale Cochrane reviews indicated that there is a high certainty that (1) nicotine-containing e-cigarettes increase cessation rates compared to NRT [9], and (2) nicotine-containing e-cigarettes, nicotine patches, fast-release NRT, and bupropion are all effective for smoking cessation [10].

Use of ATPs other than e-cigarettes for smoking cessation has received less attention. For example, analysis of nationally-representative 2011 data showed that 7.8% of adults who smoked or had quit smoking within the past two years, switched to smokeless tobacco products such as chewing tobacco, snuff, or snus during their last quit attempt [4]. The study found no evidence that switching to either e-cigarettes or smokeless tobacco promoted smoking cessation [4]. In addition, little is known about whether switching to other ATPs or using evidence-based cessation aids influences switching to e-cigarettes or nicotine pouches during the same quit attempt.

Because smoking cessation often requires multiple quit attempts, it is important that adults who have an unsuccessful attempt continue their effort to quit [11–13]. This is especially critical for adults with high nicotine dependence, such as those who smoke daily, because they are at higher risk for adverse health outcomes from smoking [14]. Switching to e-cigarettes has been shown to facilitate future quitting among adults who recently quit but resumed smoking [7]. In addition, among adults who smoke cigarettes occasionally or daily, using e-cigarettes to quit smoking was positively associated with intention to quit smoking [15]. Despite the importance of these findings, there is limited understanding of whether switching to ATPs to quit is associated with quit intentions or interest in quitting among adults who smoke daily. Moreover, to our knowledge, no studies have specifically examined the potential benefits of switching to nicotine pouches, a relatively new class of tobacco products distinct from traditional smokeless tobacco products [16, 17].

In this study, we focus on adults who smoke regular cigarettes daily and have had at least one serious quit attempt, defined as an attempt lasting a day or longer, in the past 12 months. The **study objectives** pertain to quitting behaviors during the last (unsuccessful) serious quit attempt in this population and include:

1. Estimating the current prevalence of switching to ATPs and using evidence-based cessation aids. **Hypothesis 1**: Based on prior literature [2, 5–7], we hypothesize that among ATPs, the prevalence will be highest for e-cigarettes, and among all ATPs and cessation aids, the prevalence will be highest for NRT.
2. Determining whether switching to other ATPs or using evidence-based cessation aids is associated with also switching to e-cigarettes or nicotine pouches during the same quit attempt. **Hypothesis 2.1**: We hypothesize that switching to e-cigarettes or nicotine pouches will be more common among adults who also switched to other ATPs; and **Hypothesis 2.2**: Switching to e-cigarettes or nicotine pouches will be more common among those who used evidence-based cessation aids compared to those who did not.
3. Determining whether switching to ATPs, relative to using evidence-based cessation aids, is associated with increased quit intention or interest in quitting. **Hypothesis 3**: We hypothesize that, unlike use of evidence–based cessation aids, switching to ATPs will not be associated with improved quit intention or interest.

## Methods

We used the 2022-23 Tobacco Use Supplement to the Current Population Survey (TUS-CPS) data for U.S. adults aged 18 years and older. The TUS-CPS, administered by the U.S. Census Bureau in all 50 states approximately every four years, is conducted in three monthly waves per survey period (e.g., 2022-23) [18]. The study included adults who smoked regular cigarettes daily at the time of assessment and had at least one serious quit attempt (defined as an attempt lasting at least a day) in the past 12 months. The study included only respondents with complete information on all primary study measures. The samples included 3,038 respondents for addressing objectives 1 and 2; 2,962 respondents for assessing quit intention (objective 3); and 3,000 respondents for assessing interest in quitting (objective 3).

**Switching to ATPs** was assessed using binary measures (yes/no) indicating whether respondents switched during their last serious quit attempt to: (1) cigars or pipes filled with tobacco, including regular cigars, cigarillos, little filtered cigars, and any pipes filled with tobacco; (2) e-cigarettes; (3) nicotine pouches; and (4) smokeless tobacco, such as chewing tobacco, snuff, and snus. The corresponding survey items were similar for all these measures. For example, switching to e-cigarettes and switching to nicotine pouches (dependent variables in objectives 1 and 2, independent variables in objective 3) were defined using responses to the TUS-CPS questions, respectively: “The last time you tried to quit smoking in the past 12 months: Did you do any of the following: (1) Try to quit by switching to electronic or e-cigarettes? and (2) Try to quit by switching to nicotine pouches?” Switching to cigars or pipes filled with tobacco and switching to smokeless tobacco (independent variables in objectives 1—3) were defined similarly.

Additional binary measures (yes/no; dependent variables in objective 3) included: **quit intention**, differentiating adults who seriously considered quitting smoking within the next 6 months from those who did not, and **interest in quitting**, measured on a scale from 1 (not at all interested) to 10 (extremely interested).

Moreover, **use of evidence-based smoking cessation aids** (independent variables) during the last serious quit attempt was defined separately for the following aids: (1) NRT, such as patch, gum, lozenge, nasal spray, and inhaler; (2) prescription pills, such as Chantix^®^ (Varenicline), and Wellbutrin^®^ or Zyban^®^ (Bupropion); (3) quitlines (telephone helplines); (4) individual in-person counseling by a health professional; (5) group counseling or support groups, including stop smoking clinics and classes; and (6) digital tools or programs, including web-based programs/tools, smartphone apps, and text messaging programs.

The **indicator of smoking status 12 months prior to the assessment** (independent variable in objectives 1 and 2) differentiated between adults who smoked daily 12 months prior to the assessment and those who smoked occasionally or not at all. **Sociodemographic characteristics** (independent variables in objectives 1 and 2) are listed in Table 1. **Current tobacco use behaviors** and **quit attempt history**, which were used only to describe the sample and not included in subsequent analysis, are also presented in Table 1.

**Table 1:** Characteristics of adults who smoke daily and had a serious quit attempt in the past 12 Months (*n* = 3,038; *N* = 6,218,284)^a^

Characteristic	Sample Count	Weighted Percent^a^ (%)
**Sociodemographic Characteristic**		
**Age**		
18–25	86	4.72
26–44	935	34.08
45–64	1308	43.47
65+	709	17.73
**Sex**		
Men	1481	51.75
Women	1557	48.25
**Race/Ethnicity**		
Hispanic	210	10.26
Non-Hispanic Black/African American	361	13.59
Non-Hispanic White	2290	70.05
Non-Hispanic Other^b^	177	6.10
**Marital Status**		
Married (living with a spouse)	1092	37.73
Widowed, divorced or separated	1146	31.96
Never married	800	30.31
**Highest Level of Education**		
Less than high school	422	14.59
High school or equivalent	1179	41.95
Some college, bachelor’s degree, graduate degree, or equivalent	1437	43.46
**Employment Status**		
Employed	1526	53.91
Unemployed or not in labor force	1512	46.09
**Annual Family Income**		
Less than $20,000	717	19.03
$20,000 – $39,999	790	24.89
$40,000 – $74,999	835	28.09
$75,000+	696	27.99
**U.S. Region of Residence**		
Northeast	456	16.47
Midwest	766	27.37
South	1165	39.63
West	651	16.53
**Metropolitan Status (i.e.**,** Area of Residence)**		
Metropolitan area	2201	79.25
Non-metropolitan or unknown^c^	837	20.75
**Tobacco Use Behavior**		
**Indicator of Daily Smoking 12 Months Prior to The Assessment**		
Yes (i.e., smoked daily)	2565	84.24
No (i.e., smoked occasionally or not at all)	473	15.76
**Number of Cigarettes Currently Smoked per Day**		
1–9	911	31.84
10–30	2104	68.16
**Current use of E-Cigarettes** ^**d**^		
Yes (Daily or Occasionally)	224	8.39
No	2799	91.61
**Current Use of Nicotine Pouches** ^**d**^		
Yes (Daily or Occasionally)	32	1.12
No	2920	98.88
**Quit Attempt History (Past 12 Months)**		
**Number of Serious Quit Attempts** ^**e**^		
1–3	1887	74.24
4+	672	25.76
**Duration of the Last Serious Quit Attempt** ^f^		
1–6 Days	365	46.93
7 + Days	398	53.07

^a^N denotes the total population count. Percentages are based on the corresponding population counts. In addition to the characteristics listed, survey mode (phone, in-person) was considered: 54.25% of interviews were conducted in-person and 45.75% by phone. Survey mode was not significantly associated with switching to e-cigarettes or nicotine pouches in bivariate analyses; due to missing values for 17 respondents, it was not included in the further analyses

^b"^Non-Hispanic (NH) Other” group includes 60 NH American Indian/Alaska Native, 52 NH Asian/Asian American, 6 NH Hawaiian/Pacific islander, and 59 NH Multiracial adults

^c^Metropolitan status was unknown for 53 (1.29%) respondents

^d^Current use of e-cigarettes (or nicotine pouches) was defined by combining responses to two survey items: ever use (yes/no) and current use (daily, occasionally, not at all) among respondents who reported having ever used the product

^e^Number of serious quit attempts (past 12 months) was available for 2,559 respondents

^f^Duration of the last serious quit attempt (past 12 months) was available for 763 respondents

To assess objective 2 and examine quit intention (objective 3), we used Rao-Scott chi–squared tests at the 5% significance level [19]. For objective 2, we also fitted two design-specific multiple logistic regression models: one for the logit of switching to e-cigarettes to quit smoking (versus not switching) and the other for the logit of switching to nicotine pouches to quit smoking (versus not switching). Each model included use of all 6 evidence-based cessation aids and switching to other ATPs, and controlled for the indicator of daily smoking 12 months prior to the assessment and sociodemographic characteristics listed in Table 1. For predictors that were significant at the 5% level, a 95% simultaneous confidence band based on Bonferroni-adjusted confidence intervals (CIs) was computed. Predictors significant at the 10% level were also noted (see Appendix). To test for differences in the mean interest in quitting score associated with switching to an ATP or use of a cessation aid (objective 3), we fitted separate design-specific simple linear regression models.

All analyses incorporated TUS-CPS survey weights and Balanced Repeated Replications with a Fay coefficient of 0.5 [20]. Analyses were conducted using the SAS^®^9.4 package [21].

## Results

### Prevalence of switching to ATPs or use of cessation aids

Table 1 presents the sample description including sociodemographic characteristics and current tobacco use behaviors. Among adults who smoked daily at the time of the assessment and had at least one serious quit attempt in the past 12 months, 84.24% smoked daily 12 months prior to the assessment and 68.16% smoked 10 or more cigarettes per day at the time of the assessment. The prevalence of switching to an ATP to quit smoking was 20.93% (SE = 1.06%) for e-cigarettes, 5.46% (SE = 0.51%) for nicotine pouches, 3.90% (SE = 0.49%) for cigars and pipes filled with tobacco, and 3.41% (SE = 0.42%) for smokeless tobacco. [These percentages are based on marginal distributions and therefore include adults who also switched to other ATPs.] The prevalence of using an evidence-based cessation aid was 32.10% (SE = 1.10%) for NRT, 11.75% (SE = 0.73%) for prescription pills, 4.06% (SE = 0.49%) for quitlines, 5.71% (SE = 0.52%) for individual in-person counseling, 2.29% (SE = 0.31%) for group counseling or support groups, and 2.41% (SE = 0.32%) for digital tools or programs. [The highest prevalence of co-use of NRT with a behavioral aid was 3.21% (SE = 0.44%) for NRT and quitlines, followed by 3.06% (SE = 0.37%) for NRT and in-person counseling, and 1.48% (SE = 0.25%) for NRT and group counseling/support groups.] These results are consistent with **Hypothesis 1**, which stated that, among ATPs, e-cigarettes would have the highest prevalence, and among cessation aids and ATPs, NRT would have the highest prevalence.

### Prevalence of switching to e-cigarettes and nicotine pouches: differences by use of other ATPs or cessation aids

Table 2 presents the prevalence of switching to e-cigarettes and nicotine pouches to quit smoking, stratified by whether adults switched to another ATP and by whether they used an evidence-based cessation aid (comparisons were based on Rao-Scott chi–squared tests).

**Table 2:** Prevalence (and Standard Error, %) of switching to e-cigarettes and nicotine pouches to quit smoking by use of ATPs and cessation aids (*n* = 3,038; *N* = 6,218,284)^a^

ATP or Cessation Aid	Prevalence (%) of Switching to E-Cigarettes to Quit Smoking			Prevalence (%) of Switching to Nicotine Pouches to Quit Smoking		
	Used the ATP or Aid	Did Not Use the ATP or Aid	*p*-Value	Used the ATP or Aid	Did Not Use the ATP or Aid	*p*-Value
**ATP**						
Cigars and pipes filled with tobacco	45.75 (6.80)	19.92 (1.05)	< 0.0001	23.15 (5.50)	4.74 (0.44)	< 0.0001
E-cigarettes				10.56 (1.64)	4.11 (0.50)	< 0.0001
Nicotine pouches	40.48 (4.95)	19.80 (1.10)	< 0.0001			
Smokeless tobacco	53.60 (6.40)	19.77 (0.99)	< 0.0001	37.11 (5.52)	4.34 (0.46)	< 0.0001
**Cessation Aid**						
NRT	26.35 (1.81)	18.36 (1.28)	0.0002	13.04 (1.24)	1.87 (0.40)	< 0.0001
Prescription pill	26.33 (2.70)	20.21 (1.12)	0.0215	10.90 (2.01)	4.73 (0.50)	< 0.0001
Quitline	34.04 (4.57)	20.37 (1.10)	0.0009	19.40 (5.04)	4.87 (0.46)	< 0.0001
Individual in-person counseling	28.72 (4.35)	20.45 (1.09)	0.0410	10.33 (2.93)	5.16 (0.50)	0.0199
Group counseling or support group	37.68 (7.42)	20.53 (1.07)	0.0062	14.29 (4.67)	5.25 (0.50)	0.0028
Digital tool or program	35.92 (6.50)	20.56 (1.07)	0.0054	25.02 (6.25)	4.98 (0.47)	< 0.0001

^a^N denotes the total population count. Prevalence is based on the corresponding population count. P-values correspond to Rao-Scott chi–squared tests

Table 3 presents key results from the models for switching to e-cigarettes and nicotine pouches. Only significant predictors related to switching/use behaviors are shown. The indicator of daily smoking 12 months prior to the assessment was not significant in either model. Additional information, including model fit statistics, is presented in the Appendix.

**Table 3:** Key results from multiple logistic regression models: adjusted odds ratio (aOR) and 95% confidence interval (CI)

ATP or Cessation Aid Used (Yes vs. No)	Switching to E-Cigarettes to Quit Smoking			Switching to Nicotine Pouches to Quit Smoking		
	aOR	95% CI	*p*-Value	aOR	95% CI	*p*-Value
Cigars and pipes filled with tobacco	2.376	1.263–4.471	0.0073	3.013	1.371–6.622	0.0060
E-cigarettes				1.741	1.034–2.931	0.0371
Smokeless tobacco	2.923	1.614–5.295	0.0004	7.331	3.420-15.718	< 0.0001
NRT	1.503	1.133–1.994	0.0047	6.337	3.785–10.608	< 0.0001

These results support **Hypothesis 2.1**, which stated that switching to e-cigarettes or nicotine pouches would be more common among adults who also switched to other ATPs. However, after adjusting for other factors, use of NRT was significantly (positively) associated with both switching to e-cigarettes and switching to nicotine pouches. Therefore, the results provided evidence contrary to **Hypothesis 2.2.**

### Intention to quit smoking within 6 months and interest in quitting

Overall, 66.32% (SE = 1.12%) of adults reported that they intend to quit smoking within the next 6 months. The interest in quitting scores ranged from 1.00 to 10.00; the overall mean score was 6.70 (SE = 0.07), and the median score was 6.83 (SE = 0.12). Table 4 shows the percentages of adults intending to quit smoking within the next 6 months and the mean interest in quitting score, stratified by switching to an ATP and use of a cessation aid. These results are based on analyses unadjusted for other characteristics; they support **Hypothesis 3**, which stated that, unlike use of evidence–based cessation aids, switching to ATPs would not be associated with improved quit intention or interest.

**Table 4:** Quit intention and interest in quitting, by use of ATPs and cessation aids

ATP or Cessation Aid	Percentage (and Standard Error, %) of Adults Who Intend to Quit (Next 6 Months) *n* = 2,962; *N* = 6,068,117^a^			Mean Interest in Quitting Score (and Standard Error) *n* = 3,000; *N* = 6,139,836^a^		
	Used the ATP or Aid	Did Not Use the ATP or Aid	*p*-Value^b^	Used the ATP or Aid	Did Not Use the ATP or Aid	*p*-Value^c^
**ATP**						
Cigars and pipes filled with tobacco	63.90 (6.57)	66.42 (1.11)	NS^d^	6.61 (0.39)	6.70 (0.07)	NS
E-cigarettes	65.85 (2.46)	66.45 (1.21)	NS	6.68 (0.17)	6.70 (0.08)	NS
Nicotine pouches	69.35 (4.22)	66.14 (1.16)	NS	6.60 (0.28)	6.70 (0.07)	NS
Smokeless tobacco	72.09 (5.66)	66.12 (1.12)	NS	6.86 (0.36)	6.69 (0.07)	NS
**Cessation Aid**						
NRT	69.62 (1.92)	64.75 (1.42)	0.0499	6.99 (0.12)	6.56 (0.09)	0.0059
Prescription pill	74.54 (2.97)	65.21 (1.18)	0.0055	7.47 (0.18)	6.59 (0.07)	< 0.0001
Quitline	79.35 (4.65)	65.77 (1.11)	0.0109	7.84 (0.32)	6.65 (0.07)	0.0004
Individual in-person counseling	75.75 (3.91)	65.75 (1.14)	0.0203	7.35 (0.25)	6.66 (0.07)	0.0091
Group counseling or support group	73.42 (5.67)	66.16 (1.13)	NS	7.53 (0.39)	6.68 (0.07)	0.0322
Digital tool or program	84.99 (4.69)	65.86 (1.14)	0.0023	8.31 (0.26)	6.66 (0.07)	< 0.0001

^a^N denotes the total population count

^b^P-values correspond to Rao-Scott chi–squared tests

^c^P-values correspond to tests of the predictor coefficient in simple survey linear regression models

^d^"NS” stands for not significant (at 5% level)

## Discussion

### Key findings

The study found that among adults who smoke daily and made a serious quit attempt (lasting a day or longer) in the past 12 months, e-cigarettes were the most commonly used ATP for quitting, followed by nicotine pouches. In this population, the prevalence of using e-cigarettes (20.93%) and nicotine pouches (5.46%) to quit, as well as their current use (see Table 1), was considerably higher than in the general adult population (3.0% and 0.5%, respectively) [18]. The higher prevalence of using e-cigarettes or nicotine pouches to quit smoking, compared to use in the general adult population, highlights their emerging role in quit attempts.

In addition, switching to smokeless tobacco products and switching to cigars or pipes filled with tobacco were positively associated with both switching to e-cigarettes and switching to nicotine pouches. Likewise, switching to e-cigarettes was positively associated with switching to nicotine pouches. Among the considered cessation aids, after adjusting for other factors, use of NRT was significantly (positively) associated with both switching to e-cigarettes and switching to nicotine pouches. These findings highlight the importance of considering diverse quit attempt characteristics and behaviors in tobacco research, rather than focusing solely on the use of evidence-based cessation aids.

Our study also shows that NRT remains the most commonly used evidence-based cessation aid and is used more frequently than switching to e-cigarettes or nicotine pouches. This aligns with prior literature indicating that, while some adults prefer nicotine pouches over NRT products such as gum or lozenges [22], NRT remains more commonly used than ATPs [5]. However, our secondary findings indicate that the co-use of NRT with a behavioral aid, a standard cessation treatment, is relatively uncommon.

Among ATPs, only switching to smokeless tobacco was associated with a higher percentage of adults intending to quit and a greater mean interest in quitting score, and switching to nicotine pouches was associated with a higher percentage of adults intending to quit; however, none of these differences were statistically significant. In contrast, use of any cessation aid, except group counseling or support groups, was positively associated with quit intention. Likewise, for all cessation aids considered, the mean interest in quitting score was significantly higher for adults who used the aid compared with those who did not. Among the cessation aids, use of digital tools or programs was associated with the highest percentage of adults intending to quit and the highest mean interest in quitting score. Thus, unlike the use of evidence–based cessation aids, which was associated with higher quit intention and interest, switching to ATPs was not associated with increased quit intention or interest.

The estimates obtained in this study are consistent with prior literature. First, the estimated prevalence of switching to e-cigarettes (21%) aligns with estimates from other studies, which range from 22% to 24% depending on the population studied [5, 7, 23]. Second, the estimated prevalence of use of evidence-based cessation aids (e.g., 32.10% for NRT) is also consistent with prior literature [24], e.g., 29% for NRT use among adults who smoked daily at the time of assessment or had quit smoking within 24 months (after previously smoking daily) [5]. Nonetheless, the study also demonstrates that cessation aids continue to be underutilized [6, 24].

### Limitations and future research directions

#### Our study has several limitations

First, switching to ATPs to quit may depend on the level of nicotine dependence, but the TUS-CPS does not survey nicotine dependence just prior to the last serious quit attempt. Therefore, we used the indicator of daily smoking 12 months prior to the assessment as a proxy measure for the nicotine dependence. The association between this indicator and switching to e-cigarettes, as well as switching to nicotine pouches, was not significant. However, this does not imply that nicotine dependence just prior to the quit attempt has no influence on ATP use. A related limitation is the lack of information on ATP use immediately prior to the quit attempt as well as not including quit attempt history in the analysis (due to sample size limitation). A more comprehensive analysis is needed to assess the role of these factors.

Second, characteristics of the e-cigarettes used to quit smoking are unknown. This is a serious limitation as evidence suggests nicotine-containing e-cigarettes may be more effective for cessation than nicotine-free e-cigarettes [9, 10].

Third, the order in which the ATPs and cessation aids were used during the same quit attempt by adults who used multiple methods is unknown. For example, the significant associations between NRT use and switching to e-cigarettes, as well as switching to nicotine pouches, do not necessarily imply that NRT use preceded or caused use of the ATPs.

Fourth, no causal relationships should be inferred from the study findings, even though the timing of events appears logical. Although quit behaviors occurred prior to current quit intentions and interest, these intentions and interest may change over time, and the ones considered in the study may differ from those immediately following the quit attempt. Moreover, reports of smoking-related behaviors are subject to potential response bias [25, 26]. In this study, respondents may have recalled methods used during their last serious quit attempt imprecisely or combined multiple quit attempts into one, particularly if the periods of smoking between attempts were brief. Future studies should assess the longitudinal patterns of use of various aids and tobacco products.

Fifth, the study did not include adults who successfully quit smoking and did not assess effectiveness of ATPs for smoking cessation or harm reduction. The finding that 4% of adults tried to quit smoking by switching to cigars or pipes filled with tobacco, which involve combustion and are not considered harm-reduction tobacco products, reinforces the importance of future investigations of perceptions and choices made by adults who smoke. For example, more research is needed to understand how specific ATPs are selected during quit attempts and how these choices relate to cessation and harm reduction.

Finally, the model for the logit of switching to e-cigarettes had only a modest level of classification (with 69% of concordant observations), potentially suggesting that other important factors were not considered. Future studies should explore additional predictors of switching to e-cigarettes to quit. Moreover, future research is needed to assess the role of ATPs as a harm reduction strategy among adults who smoke heavily.

## Conclusions

While use of evidence-based cessation aids during the most recent serious, unsuccessful quit attempt was positively associated with quit intention and interest in quitting, switching to ATPs during the attempt was not. These findings provide important evidence to inform future research on tobacco cessation behaviors. Moroever, in light of recent evidence that use of nicotine-containing e-cigarettes could support cessation, further research is needed to determine whether ATPs could be beneficial, e.g., for adults experiencing a relapse after using evidence-based smoking cessation aids.

## Supplementary Information

Below is the link to the electronic supplementary material.

Supplementary Material 1

## Data Availability

The TUS-CPS data are publicly available. The study dataset and SAS^®^ 9.4 software code can be found in the Harvard Dataverse repository (Soulakova, J. 2026. Replication Data for “Switching to Alternative Tobacco Products During Unsuccessful Quit Attempts Is Not Linked to Improved Quit Intention or Interest.” Harvard Dataverse. https://doi.org/10.7910/DVN/OMQUDS).

## Abbreviations

ATP: Alternative tobacco product
NRT: Nicotine replacement therapy
TUS: CPS–Tobacco Use Supplement to the Current Population Survey
