# Supplementary material for: Switching to alternative tobacco products during unsuccessful smoking quit attempts is not linked to improved quit intention or interest
Source: Addict Sci Clin Pract. 2026 May 25;21:46. doi: 10.1186/s13722-026-00674-2 (PMC13217819; doi:10.1186/s13722-026-00674-2)
Supplement: Supplementary file 1 — Supplementary Material 1 [file 13722_2026_674_MOESM1_ESM.docx]

**APPENDIX**

This appendix contains supplemental model-assisted analyses provided to support the results presented in the main text.

**The model for the logit of switching to e-cigarettes** was significant (Likelihood Ratio Chi-Square ≈ 540,328; df=29, p<0.0001; n=3,038; N= 6,218,284; 69% of concordant and 31% of discordant observations). The model indicated that the prevalence of switching to e-cigarettes was significantly higher among adults who switched to cigars or pipes filled with tobacco (aOR=2.376; 95%CI=1.263:4.471; p=0.0073), among adults who switched to smokeless tobacco products (aOR=2.923; 95%CI=1.614:5.295; p=0.0004), and among adults who used NRT (aOR=1.503; 95%CI=1.133:1.994; p=0.0047) relative to those who did not switch/use the product/aid. The other significant predictors in the model were age group (p<0.0001) and race/ethnicity (p=0.0046). The prevalence of switching to e-cigarettes was significantly higher among 18-25 year-old adults (aOR=5.799, 98.33%CI=2.326:14.455), 26-44 year-old adults (aOR=2.992, 98.33%CI=1.718:5.212), and 45-64 year-old adults (aOR=1.647, 98.33%CI=1.052:2.579) relative to 65+ year-old adults, and significantly lower among non-Hispanic Black/African American adults relative to non-Hispanic White adults a(OR=0.520, 98.33%CI=0.323:0.838). The other racial/ethnic differences were not significant.

In addition, switching to nicotine pouches (p=0.0613), participating in a group counseling or support group (p=0.0984), sex (p=0.0642), and metropolitan status (p=0.0505) were significant at the 10% level.

The indicators of use of prescription pills, quitlines, individual in-person counseling, or digital tools or programs; indicator of daily smoking 12 months prior to the assessment; and marital status, educational attainment, employment status, income, and U.S. region of residence were not significant.

**The model for the logit of switching to nicotine pouches** was significant (Likelihood Ratio Chi-Square ≈ 612,763; df=29, p<0.0001; n=3,038; N=6,218,284; 81% of concordant and 18% of discordant observations). The model indicated that the prevalence of switching to nicotine pouches was significantly higher among adults who switched to cigars or pipes filled with tobacco (aOR=3.013; 95%CI=1.371:6.622; p=0.0060), e-cigarettes (aOR=1.741; 95%CI=1.034:2.931; p=0.0371), smokeless tobacco products (aOR=7.331; 95%CI=3.420:15.718; p<0.0001), and among adults who used NRT (aOR=6.337; 95%CI=3.785:10.608; p<0.0001) compared with those who did not switch/use the product/aid.

In addition, using digital tools or programs (p=0.0925) and age group (p=0.0770) were significant at the 10% level.

The indicators of use of prescription pills, quitlines, individual in-person counseling, and use of group counseling or support groups; sex, race/ethnicity, marital status, educational attainment, employment status, income, U.S. region of residence, and metropolitan status; and the indicator of daily smoking 12 months prior to the assessment were not significant.
